# High-resolution structure of the presynaptic RAD51 filament on single-stranded DNA by electron cryo-microscopy

**DOI:** 10.1093/nar/gkw783

**Published:** 2016-09-05

**Authors:** Judith M. Short, Yang Liu, Shaoxia Chen, Neelesh Soni, Mallur S. Madhusudhan, Mahmud K.K. Shivji, Ashok R. Venkitaraman

**Affiliations:** 1 Medical Research Council Cancer Unit, University of Cambridge, Hills Road, Cambridge CB2 0XZ, UK; 2 Medical Research Council Laboratory of Molecular Biology, Francis Crick Avenue, Cambridge CB2 0QH, UK; 3 Indian Institute of Science, Education & Research, Dr Homi Babha Road, Pune 411 008, India; 4 Bioinformatics Institute, A*STAR, 30 Biopolis Drive, 138671 Singapore

## Abstract

Homologous DNA recombination (HR) by the RAD51 recombinase enables error-free DNA break repair. To execute HR, RAD51 first forms a presynaptic filament on single-stranded (ss) DNA, which catalyses pairing with homologous double-stranded (ds) DNA. Here, we report a structure for the presynaptic human RAD51 filament at 3.5–5.0Å resolution using electron cryo-microscopy. RAD51 encases ssDNA in a helical filament of 103Å pitch, comprising 6.4 protomers per turn, with a rise of 16.1Å and a twist of 56.2°. Inter-protomer distance correlates with rotation of an α-helical region in the core catalytic domain that is juxtaposed to ssDNA, suggesting how the RAD51–DNA interaction modulates protomer spacing and filament pitch. We map Fanconi anaemia-like disease-associated RAD51 mutations, clarifying potential phenotypes. We predict binding sites on the presynaptic filament for two modules present in each BRC repeat of the BRCA2 tumour suppressor, a critical HR mediator. Structural modelling suggests that changes in filament pitch mask or expose one binding site with filament-inhibitory potential, rationalizing the paradoxical ability of the BRC repeats to either stabilize or inhibit filament formation at different steps during HR. Collectively, our findings provide fresh insight into the structural mechanism of HR and its dysregulation in human disease.

## INTRODUCTION

Homologous DNA recombination (HR) to repair dsDNA breaks (reviewed in 1) begins with the 5′ to 3′ endonucleolytic resection of DNA ends, generating a long, overhanging 3′ tail that may extend for hundreds of nucleotides. The abundant ssDNA-binding protein, replication protein A, transiently engages the single-stranded 3′ tail, but is replaced by RAD51, whose nucleation on ssDNA is stabilized by the evolutionarily conserved BRC repeat motifs within the BRCA2 tumour suppressor protein (2–6). RAD51 bound to adenosine triphosphate (ATP) arranges itself on ssDNA into a dynamic, helical nucleoprotein filament—the presynaptic filament—that invades duplex DNA to search for a sequence homologous to the ssDNA tail. Strand invasion and homologous pairing via the presynaptic RAD51 filament initiate the strand synthesis and exchange events that lead to error-free repair (7,8). ATP hydrolysis by the catalytic activity of RAD51 allows the components of the repair reaction to dissociate (9,10).

The central role played by RAD51 in HR reactions has sparked much interest in its structure. Crystal structures have been reported for RAD51 (11) as well as its orthologues from simpler organisms, including RecA from *Escherichia coli* (12–14), RadA from *Pyrococcus furiosus* (15) and Rad51 from *Saccharomyces cerevisiae* (16). These proteins spontaneously oligomerize even in the absence of DNA to form rings with 6 or more protomers, or helical structures exhibiting varied symmetry. Protomers of RAD51 and its orthologues share a structurally conserved, ATP-binding core catalytic domain. In addition, archaeal RadA and yeast or human RAD51 possess a flexible amino (N)-terminal region ranging from ∼100–190 residues that participates in the protomer–protomer interface (11,15,16), and has been implicated in DNA binding (17). RecA lacks this N-terminal region, but its catalytic domain instead extends into a carboxyl (C)-terminal region bearing limited sequence homology to its archaeal or eukaryotic orthologues (12–14).

There is limited structural information concerning the assembly of ATP-bound RAD51 on ssDNA into an active presynaptic filament. Electron microscopy (EM) reveals considerable variation in helical rise and twist between RecA and RAD51 filaments under different conditions (Table 1), suggestive of conformational polymorphism and posing challenges for structural resolution. Models of RecA or RAD51 assemblies on DNA (18–20) at relatively low resolutions from 16–25Å using EM suggest that active, ATP-bound filaments exhibit an extended architecture relative to normal B-DNA, whereas, in contrast, inactive Adenosine diphosphate (ADP)-bound filaments are relatively compact. Crystal structures have been reported for helical assemblies comprising ADP-bound RecA oligomers without DNA exhibiting a pitch of 83Å incorporating 6 protomers per turn (14), pre-formed fusion proteins of RecA bound to ssDNA with a pitch of 94Å incorporating 6.1 protomers per turn (12) and a filament formed by an N-terminally truncated form of yeast Rad51 bearing a Ile345Thr mutation in the catalytic domain in an extended conformation with a pitch of 130Å incorporating 6 protomers per turn (16). Although the gain-of-function Ile345Thr mutation in yeast Rad51 confers increased DNA binding (21), and this mutant yeast Rad51 was crystallized in the presence of DNA, the structure obtained lacks observable DNA (16). Interestingly, this latter structure—thus far providing the highest resolution view of a eukaryotic RAD51 filament albeit lacking DNA—suggests that alternate protomer–protomer interfaces in the extended filament are structurally distinct (16).

**Table 1.** Summary of helical symmetry parameters for RecA and RAD51 filaments

Structure	Data collection method (resolution)	Pitch in Å	Protomers/turn
RAD51/ssDNA (this report)	EM/Cryo (4.2Å)	103	6.4
RAD51/ssDNA (19)	EM/stain	99	6.39
ScRad51/no DNA (16)	Crystallography (3.25Å)	130	6
ScRad51/dsDNA (19)	EM/stain	94	6.28
RecA (14)	Crystallography (2.3Å)	82.7	6
RecA/ssDNA (12)	Crystallography (2.8Å)	93.96	6.16
RecA/dsDNA (19)	EM/stain	91	6.16
RecA/ssDNA (20)	EM/stain (27Å)	80	6.1

The helical symmetry parameter values of the recombinases have been found to vary both between and within structures, depending to some degree as to whether they are unbound, bound to ssDNA or to dsDNA. All structures incorporate 6–6.4 protomers per turn, enabling derivation of helical twist and rise from the values shown. The first entry refers to our RAD51 presynaptic structure reported here. A low-resolution EM structure of human RAD51 by staining has a pitch of 99Å, compared to the 103Å for the high-resolution structure reported here. This difference may be due to duplexing of the DNA in the stain structure; the 99Å pitch reported for this model matches that of a RAD51/dsDNA model that we have recently computed (data not shown). The extended pitch of the ScRad51 filament may stem from the hyper-functional nature of the mutant yeast Rad51 protein and/or the absence of DNA.

Here, we have used electron cryo-microscopy followed by an image processing technique that couples maximum likelihood single particle processing methods with helical symmetry software (22–24) to calculate a three-dimensional (3D) model of the human RAD51 presynaptic filament to a near-atomic resolution of 3.5–5Å, revealing previously unrecognized structural features.

## MATERIALS AND METHODS

### Protein expression and purification

Full-length human RAD51 was cloned into the pET11d vector (Novagen), expressed in recA- BLR (DE3) competent cells (Novagen) and purified with the same method described previously (25). Aliquots of protein samples were flash-frozen in liquid nitrogen and stored until use at −80°C.

### Specimen preparation

ϕX174 ssDNA was purchased from Sigma. Rad51-ssDNA nucleoprotein filaments were formed (Supplementary Figure S1) by incubating human (Hs)RAD51 protein (5.5 μM) with φX174 72mer ssDNA (15 μM) in the buffering containing 25 mM Tris-acetate pH7.0, 100 mM NaCl, 2mM Mg^2+^, 15 μM ssDNA, 2 mM adenosine 5′-(β,γ-imido)triphosphate (AMP-PNP) and 1 mM dithiothreitol (DTT) at 37°C for 15 min.

### Cryo-EM data acquisition

Specimens for EM were prepared by application of 2 μl drops of the RAD51–ssDNA complex solution to glow-discharged holey carbon Quantifoil EM grids (R1.2/1.3), followed by blotting and rapid freezing by using FEI's Vitrobot (Mark IV) at the temperature of 4°C and 100% relative humidity. Samples were rapidly frozen using liquid ethane cooled by liquid nitrogen. Grids were transferred into cartridges for an FEI Titan-Krios electron cryo-microscope. Cartridges were loaded into the cassette and transferred to the Krios specimen stage through the Autoloader cryo-transfer. Low dose images were recorded on a Falcon II CMOS direct electron detector at 300keV and with magnification 104 478× (59 000× nominal magnification). The pixel size of the Falcon detector is 14 μm; this gave the final sampling rate as 1.34Å/pixel. The electron dose rate on the specimen was set as 20 electrons per Å^2^ per second. Objective lens defocus values were ranged from −1.5 to −4.0 μm. For frame capture 16 video frames per second from Falcon II detector were recorded. The images were collected manually or automatically using FEI EPU software. The frozen specimen on the EM grids were maintained at −170°C during the whole process of loading and image recording in the microscope column.

### Image processing

#### Calculating a starting model

Filaments were selected manually from the micrograph images using the filament boxing option in Ximdisp (26) and coordinates of the centre of each box were stored in a file. Segments with an inter-box distance corresponding to the subunit rise of 16.1Å were cut as boxes of size 200 × 200 pixels and stored as stacks of images. In order to minimize interpolation errors introduced by vertical alignment, filament angles were stored in a separate file, one angle for each segment in the corresponding order. A stack of 99928 segments was corrected for defocus by dividing by the contrast transfer function up to the first maximum, then multiplying. This has the effect of improving alignment by preserving the effect of low frequency data while applying appropriate signal-to-noise weighting for high frequency data. The corrected stack was low pass filtered to 5Å, normalized and masked with a soft rectangular mask. From this stack a 3D model was calculated using a modified version of Interative Helical Real Space Reconstruction (IHRSR) (22) starting from a simple cylinder to prevent model bias. Convergence was achieved after 12 iterations to an angular displacement of 56.2° with an axial displacement of 16.1Å (Supplementary Figure S2).

#### Classification and refinement

The 3D model calculated by IHRSR was used as a reference model. Within RELION (23), defocus values were recalculated by ctffind3 (27). A total of 171 801 segments from 6046 filaments selected from 667 micrographs were extracted with a box size of 200. Two-dimensional class averaging was carried out to 50 classes and 140 745 images selected for further processing. 3D classification to five models revealed that 83% of the segments matched model #2 after 25 iterations (Supplementary Figure S2). This 3D model was symmetrized using the IHRSR programs hsearch_lorentz and himpose. Due to noise in the model extremities, the two ends of the model were excluded from the symmetry search by a soft-edged mask. The stack of segments was refined against the symmetrized model and reached a resolution of 8.2Å. Post-processing was carried out to a resolution of 6.3Å. New movie data was collected, 69 843 segments selected from 5099 filaments from 500 micrographs. It is estimated that ∼180 000 unique RAD51 protomers were included in the dataset. The stack of segments was defocus-corrected as before, then motion-corrected (28) before 3D classification and refinement, using the 8.2Å model as a reference. Particle polishing was then carried out in RELION and 3D classification and refinement iterated with increasing values of the regularization parameter T. Local defocus values were then calculated and applied using Gctf (29). Finally, post-processing with sharpening using a b-factor of −250 was carried out to a ‘gold standard’ Fourier shell correlation (FSC) resolution of 4.2Å (Figure 2B), as previously described (30,31). This was confirmed by RESMAP (32) (Figure 2A), with a PDB to model FSC of 4.1Å at 0.4 (Figure 2C), and the PDB cross-validated to the two half-maps (33) showing almost no overfitted noise (Figure 2D).

### Fitting crystal structure coordinates to the model

Individual crystal structures were manually placed into our 4.2Å cryo-EM map and rigid-body refinement carried out using Chimera (34). Further fitting was performed in COOT (35). PDB codes of the fitted structures are: 1SZP (16) and 1N0W (11). Starting from poly-Ala, we replaced glycines and prolines and were able to fit 37 other side-chains into the density. The model was manually inspected and disordered regions were removed. The model PDB coordinates including the bound ssDNA were further refined in REFMAC (36). The Ramanchandran plot indicates that >95% of residues are within the preferred and allowed regions, with <5% outliers (Supplementary Figure S3).

### Comparative modelling of an inactive RAD51 filament from an ADP-RecA template

A homology model for the inactive counterpart of our structure for the active presynaptic RAD51 filament bound to the ATP analogue AMP-PNP (target) was constructed as per our previously reported approach (37) using the MODELLER version 9.14 suite of programmes (38,39). The structure of the *E. Coli* RecA in the inactive ADP-bound form (PDB: 1XMS, ref. 40) was used as the template. The target-template alignment was obtained by structurally superimposing the coordinates of a RAD51 monomer from our filament structure with ADP-bound RecA using Chimera (34), omitting ∼99 residues from the N-terminus where our structure deviates considerably from the RecA template. Five models were constructed using the refine.very_slow option in MODELLER, which ensures accurate energy minimization of the models. The models were assessed using the DOPE statistical potential (41), and that with the best DOPE score was chosen to represent the inactive RAD51 filament.

### Comparative modelling of BRC4 bound to an inactive RAD51 filament

To model BRC4 binding with the model for an inactive RAD51 filament, the monomeric RAD51-BRC4 crystal structure (PDB:1N0W, ref. 11) and inactive RAD51 filament model were used as templates in MODELLER. Protocols for model building and assessment were as described above. Again, five models were examined and the best one was chosen using the DOPE statistical potential.

## RESULTS AND DISCUSSION

### Validation of the EM map and fitted PDB coordinates

Sample preparation for full-length human RAD51 assembled on ssDNA in the presence of the ATP analogue, AMP-PNP, with methods for electron cryo-microscopy, are described elsewhere (‘Materials and Methods’ section, Supplementary Figure S1). Briefly, filaments from 500 EM micrograph images (Supplementary Figure S1) were analysed by a combination of helical and single-particle packages (Supplementary Figure S2), similar to that previously described (42), to create the final model.

The helical pitch of 80% of the filaments lies between 95 and 110Å (Supplementary Figure S4), with an average of 103Å. Pitch values ranged from 70 to 140Å in our samples, consistent with the previously reported structural polymorphism of RAD51 filaments. The average pitch of 103Å is closer to the 94Å pitch reported for the active RecA filament on ssDNA (12), than the 130Å pitch reported for a mutant form of yeast Rad51 without DNA (16), and concordant with previous measurements of active filaments (Table 1).

**Figure 1.**
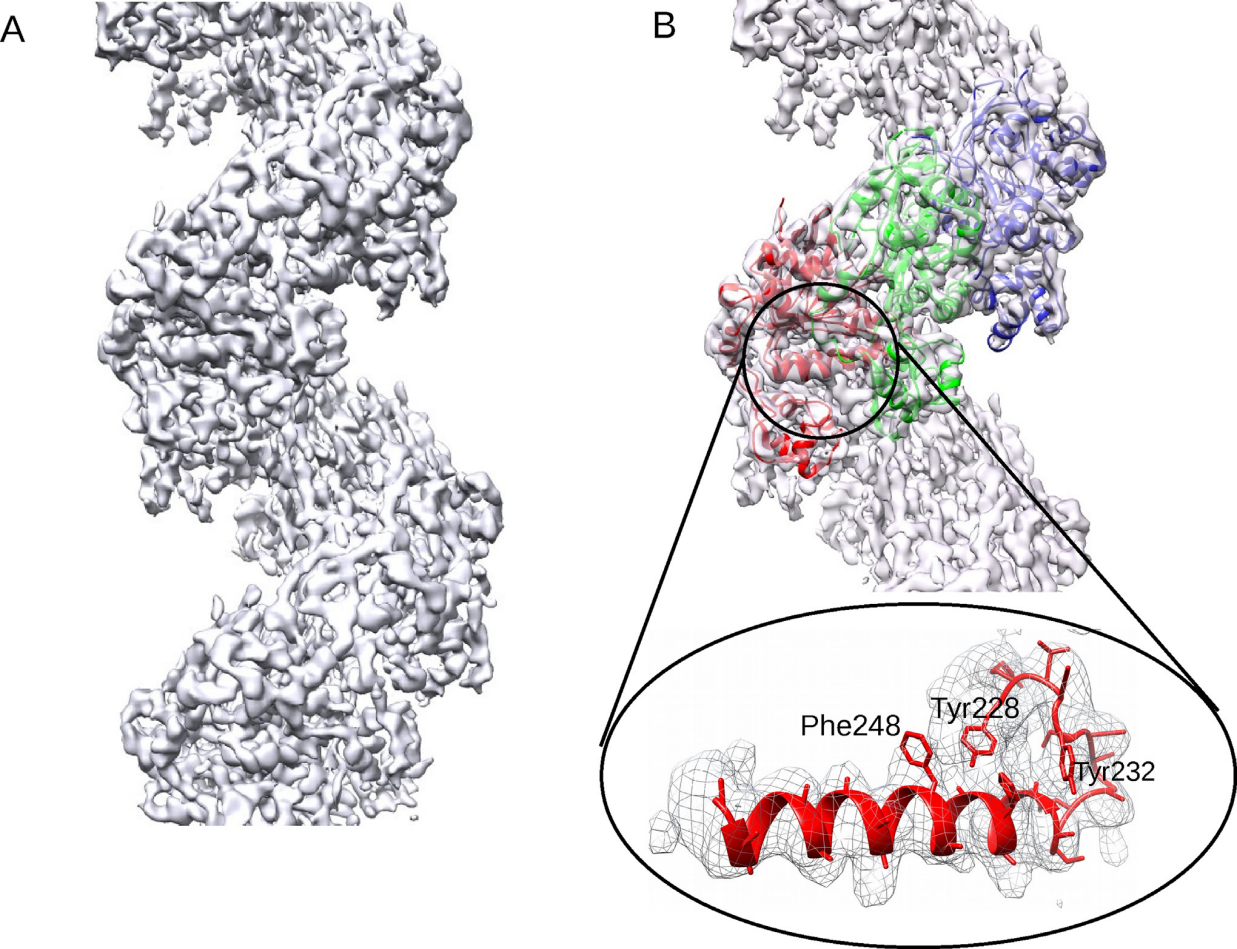
A high-resolution map of the human RAD51 presynaptic filament. (**A**) The map computed to 3.5–5Å resolution. (**B**) Backbone PDB coordinates of three monomers coloured in red, green and blue fitted into the EM map. The enlarged detail of the large α-helix that makes contact with ssDNA is in the same orientation as the red protomer from the full map, and includes fitted sidechains.

Our EM map for the presynaptic human RAD51 filament is shown in Figure 1A; α-helical regions, β-sheets and certain bulky amino acid side chains can be clearly visualized, with few regions of local disorder. We combined PDB coordinates from the structure of yeast Rad51 (PDB: 1SZP) or the core catalytic domain of human RAD51 (PDB: 1N0W) to create a single monomer of RAD51. Three individual copies of this monomer were manually placed into the EM map in Chimera (34), before further rigid-body fitting in COOT (35) and refinement in REFMAC (36). The model was manually inspected and the disordered regions were removed, until outliers in the Ramachandran plot were <5% (Supplementary Figure S3). Figure 1B shows the PDB backbone coordinates of three monomers fitted into the EM map, with the enlarged area below illustrating the **α**-helix that makes contact with the DNA along with certain visualized amino acid side-chains.

**Figure 2.**
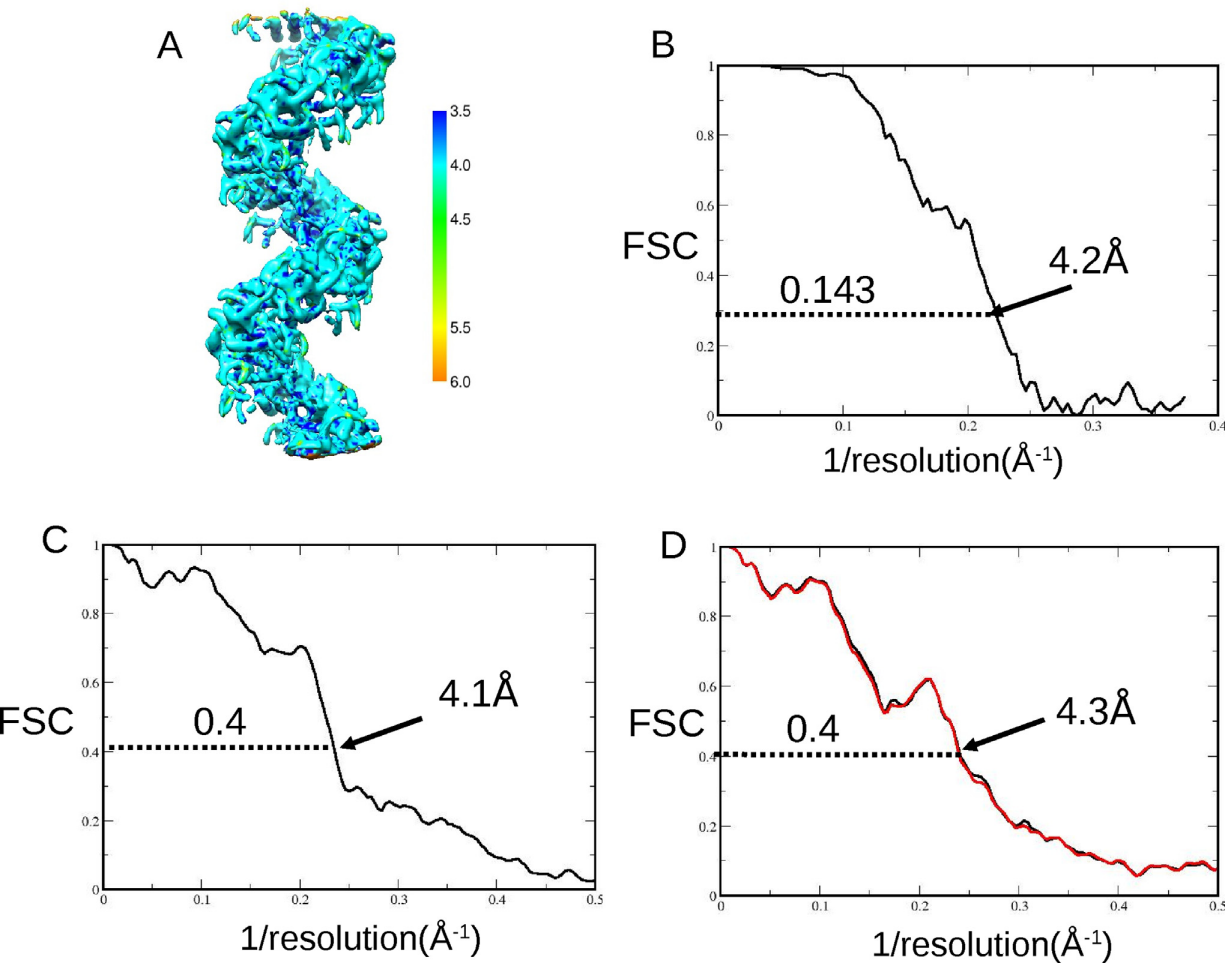
Resolution of the map and the fitted PDB coordinates with overfitted noise estimation. (**A**) Local resolution map calculated by RESMAP (32) shows the resolution range across the map from 3.5Å to 5Å. (**B**) Plot showing the Fourier shell correlation (FSC) indicating a final average map resolution of 4.2Å at a cutoff of 0.143 as calculated in RELION by the ‘gold-standard’ technique (30). (**C**) Plot showing an FSC between a masked, unfiltered area of our EM density map and an EM-derived HRad51 fitted PDB of three monomers with DNA. The cutoff of 0.4 now generally used (e.g. 31) in place of the earlier value of 0.5 indicated a fit good to 4.1Å between the fitted PDB coordinates and the EM density map. (**D**) Cross-validation to estimate the amount of overfitted noise was carried out. This works by firstly ‘shaking’ the refined PDB coordinates randomly by 0.5Å before refinement in REFMAC then conversion to EM density. FSC are computed between the new ‘shaken’, refined and converted EM density and each of the half maps from the refinement step in RELION (33). The plot shows the black line from half map 1 is very close to the red line from half map 2, indicating that there is little or no overfitted noise in our structure. The cutoff at 0.4 is shown at 4.3Å.

Several approaches were used to validate the resolution of our EM map, and the fitting of PDB coordinates. Calculation of a local resolution map (32) showed resolution ranging from 3.5 to 5.0Å (Figure 2A), while the previously reported ‘gold-standard’ FSC criterion (30) indicated an average resolution of 4.2Å at a cutoff level of 0.143 (Figure 2B). The fitted PDB coordinates, refined in REFMAC (36) were validated by FSC to 4.1Å (Figure 2C). Overfitted noise was minimal (Figure 2D) as measured by the ‘shaken’ PDB method (33).

### Architecture of the human RAD51 presynaptic filament

**Figure 3.**
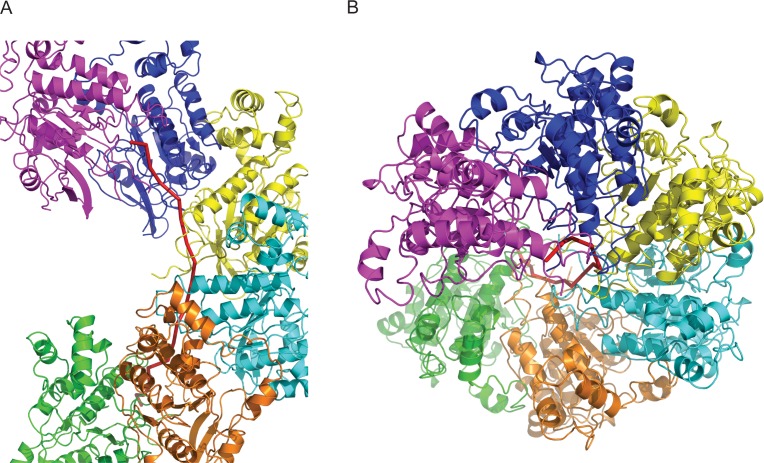
Overall architecture of the human RAD51 presynaptic filament. (**A**) The structure of the filament with 6 protomers and ssDNA colour-coded. (**B**) The top-down view of (A).

The overall architecture of the human RAD51 presynaptic filament with 6 protomers docked into the EM map when viewed perpendicular (Figure 3A) or longitudinal (Figure 3B) to the filament axis reveals that like RecA (12), human RAD51 protomers form a chain that encases ssDNA as a 1-start helix to create the presynaptic filament. The angular displacement (twist) between protomers in the presynaptic RAD51 filament is constant at 56.2°, encompassing 6.4 protomers per turn of the helix. ssDNA (red ribbon) lies between the inner surface of the protomers and the filament axis.

**Figure 4.**
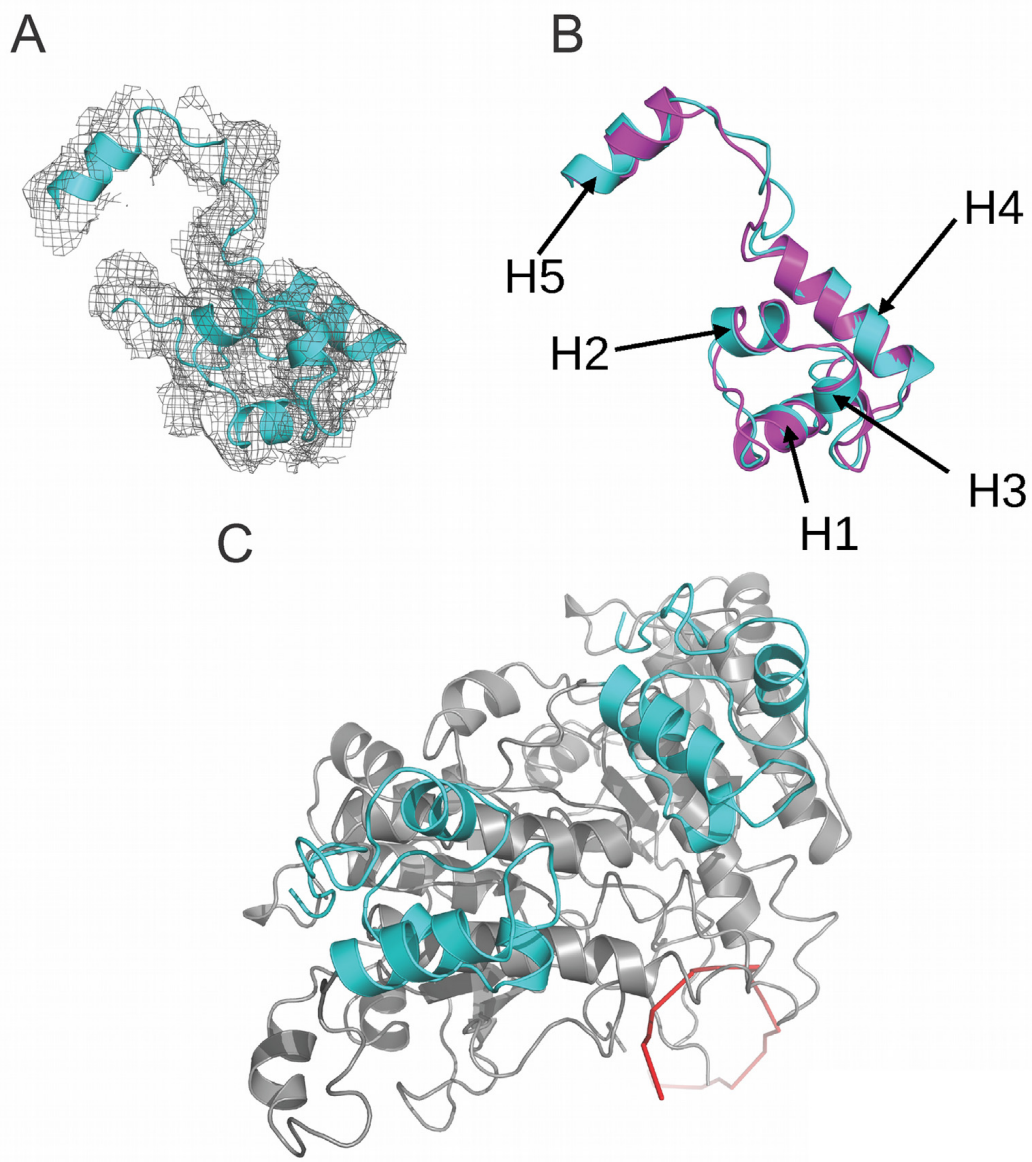
The structure of the N-terminal domain (NTD) of RAD51. (**A**) The N-terminal domain of RAD51 within the density. (**B**) Superposition of the NTD of human RAD51 (in cyan) with that of yeast Rad51 (PDB:1SZP in pink). Helices 1–5 are indicated by arrows. (**C**) The top-down view showing the two NTDs (cyan) from two adjacent protomers (in grey) against the ssDNA (in red ribbon).

The flexible N-terminal extensions of eukaryotic RAD51 proteins range from ∼100 residues in human RAD51, to ∼190 residues in yeast. A structure for the N-terminal region for yeast Rad51 has been resolved within the crystal structure of a native filament formed by an N-terminally truncated yeast Rad51 without DNA (16). This truncation removes the the first 79 residues of yeast Rad51, most of which are absent from human RAD51, but leaves ∼100 residues that correspond to the N-terminal region of human RAD51. Moreover, the N-terminal region of human RAD51 has itself been resolved in isolation (17) using nuclear magnetic resonance, or by homology modelling (43) to yeast Rad51. Our model for the N-terminal region of human RAD51 fitted to the EM density map of the filament (Figure 4A) shows a bundle of four α-helices (H1-H4) packed around a central core, with a further α-helix (H5) projected N-terminally. Superposition over the yeast Rad51 N-terminal region reveals an RMSD of 1.96Å (Figure 4B), significantly greater than for the core catalytic domain, arising not only from the truncation of helix H1 but also from slight differences in the position of interconnecting loops. When viewed top-down (Figure 4C) along the filament axis, the N-terminal regions are positioned peripherally relative to ssDNA (red ribbon), suggesting that they do not participate in RAD51–DNA interactions as previously proposed (17). Moreover, the linker between the N-terminal region and core catalytic domain forms a flexible loop distinct from the rigid helical conformation proposed by homology modelling (43).

To better visualize the orientation of the ssDNA in our EM density map, we fitted and compared it to PDB-derived EM density of RecA-bound ssDNA (Supplementary Figure S5). The fitting suggested that, just as in the RecA filament (12), the presynaptic human RAD51 filament engages the ssDNA backbone, exposing the nucleotide bases for homologous pairing reactions. ssDNA within our fitted model for the presynaptic filament lies close to the filament axis, and most likely binds the inner surface of RAD51 protomers via the L1 and L2 loop regions which encircle it, with each protomer making three contacts with ssDNA (Supplementary Figure S6).

Moreover, our model with fitted ssDNA suggests a stoichiometry of three nucleotides per RAD51 protomer in a filament with pitch 103Å, incorporating 6.4 protomers per turn. The same stoichiometry was observed in the RecA/ssDNA filament with pitch 94Å incorporating 6.1 protomers per turn (12); in comparison to this structure the ssDNA in our filament would be stretched to accommodate the longer pitch. This extended conformation is consistent with the helical parameters for other forms of active filaments formed by RAD51 and its homologues (summarized in Table 1). In the RecA filament, ssDNA nucleotides are arranged in triplets which assume a B-DNA-like conformation that favours homologous pairing to a dsDNA partner by Watson–Crick base pairing (12), which is compensated by additional stretching and twisting in the step between successive nucleotide triplets. Although our model cannot yet exclude alternative stoichiometries such as proposed for yeast Rad51 (16), the robustness of the fitting of RecA-bound ssDNA density in our EM map strongly suggests that three nucleotides bind each RAD51 in the presynaptic filament with a similar anisometric stretching of ssDNA.

### Structure and regulation of the protomer–protomer interface

Two different conformations of the protomer–protomer interface have been reported to alternate in a helical native filament formed by a mutant form of yeast Rad51 (16). This structure crystallizes as three identical but off-set hetero-dimers labelled A-D, B-C and E-F. Monomer A matches C and F while monomer D matches B and E. Although the difference between adjacent interfaces in yeast Rad51 appears to be restricted to the movement of just two side-chains (16), it does however result in a rotation of two large α-helices in the core catalytic domain. If such a feature existed in the human RAD51 presynaptic filament, symmetrizing the structure as monomeric would average the two orientations of the α-helices, and would therefore be predicted to result in a widening at one end. To ascertain whether this was true for our structure, we overlaid and fitted two copies of an unsymmetrized model, with one of the two copies helically shifted by a single protomer relative to the other. However, we observed no widening of either of the two large α-helices (marked by arrows in Supplementary Figure S7A). We also carried out 3D classification and refinement with imposed dimer symmetry; again, no observable widening of the α-helices was detected (Supplementary Figure S7B). These observations suggest that the protomer–protomer interface in the human RAD51 presynaptic filament is invariant, similar to RecA (12), but distinct from what has been proposed (16) for mutant yeast Rad51.

**Figure 5.**
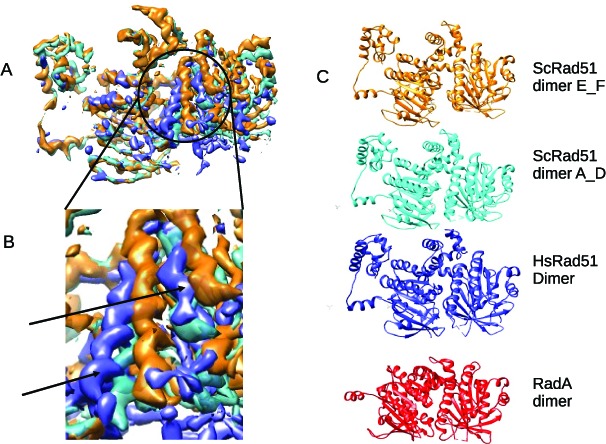
Helical pitch and protomer spacing. (**A** and **B**) Dimers A-D and E-F from the yeast Rad51 PDB coordinates (16) were converted to EM density and low-pass filtered to 8Å then fitted and overlaid in Chimera (34). This shows the rotation of the two large α-helices between adjacent A-D (turquoise) and E-F (gold) protomer–protomer interfaces. A dimer from our Rad51_ssDNA map was then overlaid (blue) (A) and shows no evidence of the widening which would be expected to accommodate rotated helices (arrowed in enlarged detail (B)). (**C**). A difference in protomer spacing can be seen between PDB coordinates (16) of yeast Rad51 dimers E-F and A-D from a filament with pitch 130Å. A dimer of PDB coordinates fitted to the EM density map of the human RAD51 presynaptic filament with pitch 103Å shows a narrower spacing than either yeast A-D or E-F. A dimer extracted from archaeal RadA PDB coordinates (15) shows even narrower spacing consistent with that of a closed ring. PDB coordinates of dimers were fitted in Chimera (34), which returned RMSD values between HsRAD51 and ScRad51 A-D as 0.976Å and between HsRad51 and ScRad51 E-F as 1.061Å.

When superimposed (Figure 5A and B) on those from yeast Rad51 (turquoise and gold), the α-helices from our model (dark blue) exhibit a rotation that brings them nearer to the adjacent protomer than does the closer spacing between protomers in the yeast Rad51 dimers A-D (turquoise) or E-F (gold). This feature corresponds to the reduced pitch for the human RAD51 presynaptic filament of 103Å, compared to 130Å for the yeast Rad51 filament lacking DNA, with each filament incorporating ∼6 protomers per turn. Indeed, the correlation (Figure 5C) between the rotation of the α-helices, closer protomer spacing and reduced filament pitch is maintained not only in the yeast Rad51 (turquoise, gold) or human RAD51 (dark blue) filaments, but also in the crystal structure of a seven-member closed ring (15) formed by *P. furiosus* RadA (red). Interestingly, the longer α-helix interacts directly with ssDNA in our model (Figure 3A and B), suggesting a mechanism whereby the RAD51–DNA interaction may modulate protomer–protomer spacing and filament pitch.

### Disease-associated RAD51 mutations

**Figure 6.**
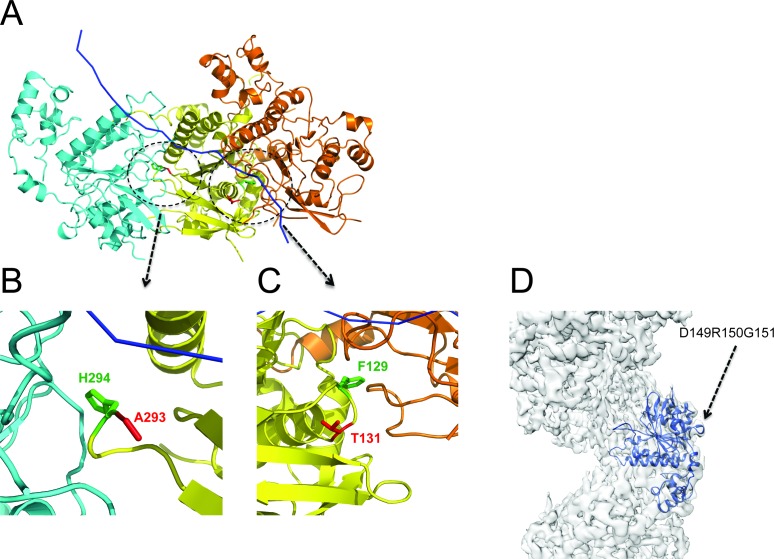
Mapping of disease-associated point mutations onto the presynaptic filament structure. (**A**) For simplicity, three adjacent protomers shown here are coloured in yellow, cyan and orange, with ssDNA shown in blue ribbon. Residues A293 and T131 are coloured in red and residue H294 and F129 in green. (**B**) A close view at the residue A293. (**C**) A close view at the residue T131. (**D**) Mapping of mutations affecting Asp149, Arg150 and Gly151 on the EM map. These residues are situated on the outer surface of the RAD51 filament, distant from the protomer–protomer interface and the ATPase sites, and are unlikely to affect filament assembly.

Two germline mutations affecting human RAD51 have recently been described in a small number of patients suffering from a Fanconi anemia-like disease (44,45), which manifests with early-onset cancer predisposition coupled to developmental anomalies such as microcephaly. One mutation alters Thr131 to Pro (T131P); Thr131 maps to the RAD51 Walker A motif implicated in ATP binding and hydrolysis. The second mutation alters Ala293 to Thr (A293T). Interestingly, both these germline mutations are heterozygous, suggesting that they work in a trans-dominant fashion to derange the normal functions of wild-type RAD51 (44,45). *In vitro* studies suggest that the T131P mutation enhances mutant RAD51 ATPase activity, and destabilizes filament formation by wild-type RAD51, while A293T impairs ATPase activity and DNA binding, and assembles with wild-type RAD51 in misshapen, unstable filaments (44,45). Mapping of T131P or A293T onto our model for the RAD51 presynaptic filament suggests structural features that may underlie their abnormal activity. In the yeast Rad51 filament, it has been suggested that Phe187 (homologous to Phe129 in human RAD51) contacts His352 (homologous to human RAD51 His294) from the adjacent protomer to stabilize the protomer–protomer interface (16). In our model for the human RAD51 presynaptic filament, both the T131P and A293T mutants map close to this interface contact (Figure 6A–C). The conformational rigidity of Pro at the 131 position is expected to distort the α-carbon backbone, which could perturb the orientation of the Phe129 side chain and its ability to contribute to the protomer–protomer interface. Substitution of Ala293 with the longer side chain of Thr may interfere with the orientation of its adjacent residue, His294, proposed to participate in the protomer–protomer interface. In both cases, mixed complexes in which mutant RAD51 interleaves with the wild-type form are expected to be less stable, and compensatory over-expression of the wild-type form (observed in a patient carrying the T131P RAD51 mutant (44,45)) could reduce the impact of the mutant protein.

Somatic RAD51 mutations affecting three adjacent residues (Asp149, Arg150 and Gly151) have recently been reported in patients with breast cancer (46). These mutations are positioned in a Schellman loop motif on the external surface of the conserved catalytic core domain, which when mapped on our model (Figure 6D), faces externally away from the protomer–protomer interface and the DNA binding region, and distant from the binding sites for BRCA2 BRC repeats. Thus, these mutations seem unlikely to directly affect RAD51 filament assembly but may instead alter its interactions with accessory proteins.

### Modelling potential interactions of the RAD51 presynaptic filament with the BRC repeats of BRCA2

Indeed, RAD51 assembly and disassembly in human cells is controlled by its physical interactions with several regulatory partners. One key partner is the breast cancer tumour suppressor, BRCA2, which performs several duties in this context. Human BRCA2 binds directly to RAD51 via eight evolutionarily conserved motifs of ∼35 residues (the BRC repeats), which are conserved in both their sequence as well as their spacing within an ∼1000 amino acid region encoded by *BRCA2* exon 11 (47). The BRCA2–RAD51 interaction promotes the localization of the protein complex to its site of function in the cell nucleus, via sequential masking of nuclear export signals that exclude the unbound protein partners (48). Within the nucleus, BRCA2 targets RAD51 to sites of DNA damage where it assembles in microscopic foci (49–53). Biochemically, the interaction of BRC repeats with RAD51 is critical in directing RAD51 filament assembly during homologous recombination. At sub-stoichiometric concentrations, BRC repeats promote the nucleation and stabilize the binding of RAD51 on ssDNA to form the presynaptic filament (2,5), whilst inhibiting the RAD51–dsDNA interaction (2,5). These opposing activities promote the stepwise completion of RAD51-mediated recombination from presynaptic filament assembly on ssDNA, to postsynaptic engagement with dsDNA (2,5). However, at higher concentrations approaching or exceeding 1:1 molar ratios *in vitro*, BRC repeats prevent RAD51 assembly on DNA, and can disassemble preformed RAD51 filaments (54).

**Figure 7.**
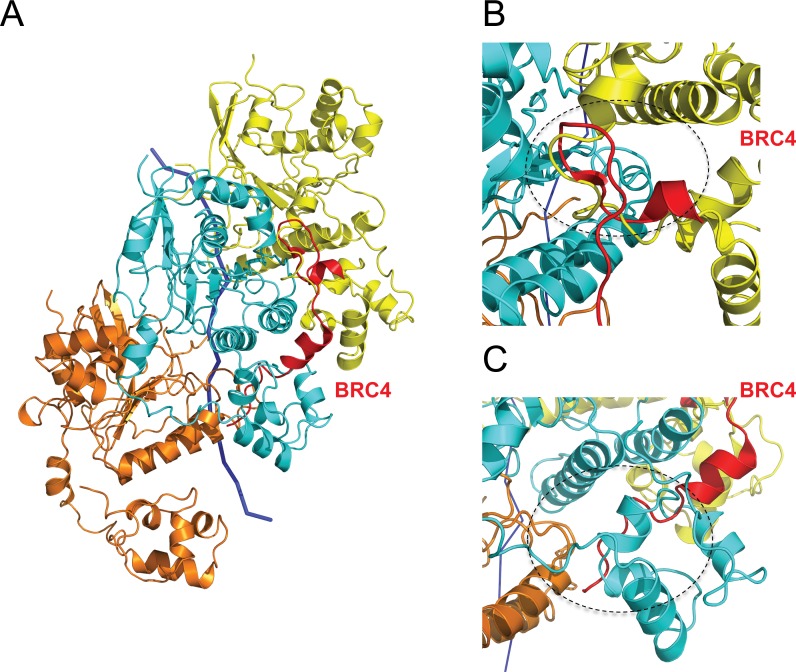
Modelling BRC4 binding to the RAD51 presynaptic filament. For simplicity, three adjacent protomers shown here are coloured in yellow, cyan and orange respectively with ssDNA shown in blue ribbon. (**A**) Two distinct modules in BRC4 (red) bind to two different sites of the presynaptic filament, one at the interface between the cyan and yellow protomers, the other between the NTD of the cyan protomer and the core catalytic domain of the orange protomer. (**B**) A close view at the interface (black dashed circle) between two adjacent RAD51 protomers (yellow and cyan) disrupted by BRC4 (red) binding. The Phe-X-X-Ala module lies adjacent to the 3:5 β-hairpin structure assumed by the BRC4 backbone (red), where it is predicted to disrupt the interface. (**C**) A close view (black dashed circle) showing the second BRC4 binding site and its predicted clash with the NTD of the cyan protomer where it clasps the adjacent orange protomer. The Leu-Phe-Asp-Glu module is positioned below the α-helical region in BRC4 (red).

The structural basis for the differing effects of BRC repeats on RAD51 assembly remains unclear. We previously solved the crystal structure (PDB: 1N0W) of a monomeric fusion between the human BRC4 repeat and the core catalytic domain of RAD51 (11). This structure predicts that a β-strand region surrounding the BRC4 residues Phe and Ala in the motif Phe-X-X-Ala (where X is any amino acid) structurally mimics RAD51 residues essential for the protomer–protomer interface, based on structural homology to the ADP-bound RecA filament (14). This prediction is supported when BRC4 (red) is overlaid onto three adjacent protomers (yellow, cyan and orange) in our model for the RAD51 presynaptic filament (Figure 7A). BRC4 contacts the middle RAD51 protomer (cyan). The previously observed 3–5 β-hairpin motif in BRC4 which serves to appose the Phe-X-X-Ala module against cognate residues on the core catalytic domain of RAD51 is positioned at the interface with the yellow protomer (Figure 7B), where it structurally mimics a corresponding structure from the N-terminal region of RAD51. Binding of the BRC4 repeat's Phe-X-X-Ala module at this site might therefore prevent RAD51 filament assembly, or promote filament disassembly, as suggested by experimental data (11,54,55).

**Figure 8.**
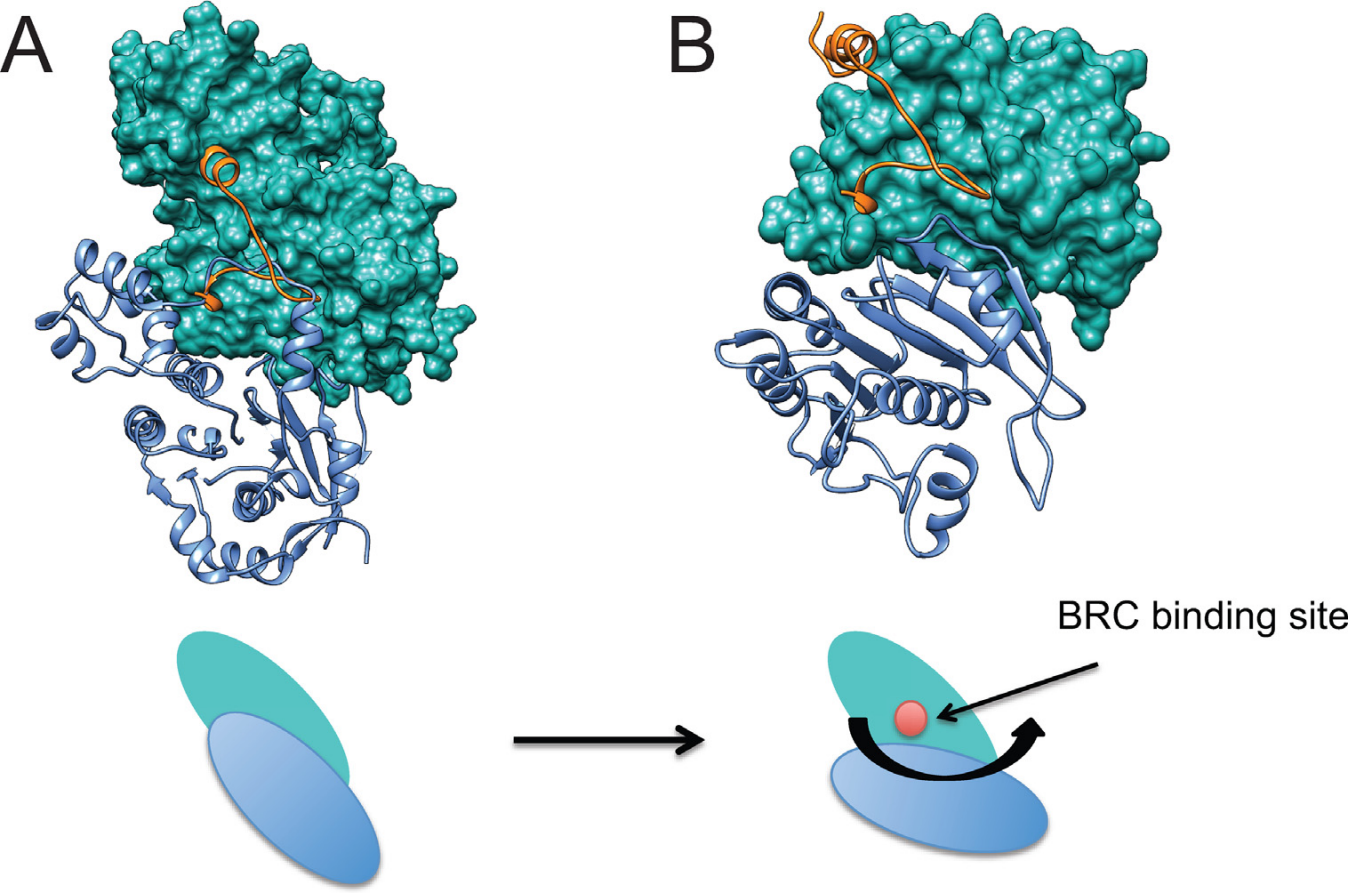
Re-orientation of RAD51 subunits during transition to an inactive filament may expose a filament-inhibitory binding site for the Phe-X-X-Ala module in the BRC repeats. Comparative modelling of the active presynaptic RAD51 filament using inactive ADP-bound RecA as a template was carried out as described in ‘Materials and Methods’ section to create a hypothetical model for an inactive RAD51 filament. Close similarity has been shown between the crystal structures of active versus ADP-bound RecA protomers (50) (RMSD < 0.8Å), and between the core catalytic domains of RecA and RAD51 (11) (RMSD < 1Å). Our modelling of the inactive RAD51 filament using RecA as a template therefore makes the minimal assumption that the structural changes underlying the active-inactive transformation are also similar. (**A**) Two protomers from our EM structure for the active presynaptic RAD51 filament are displayed, one in blue ribbon representation and another in green surface representation. A region of ∼99 residues from the N-terminus of both protomers was omitted, as described in the text, because this region is absent from RecA. BRC4 is shown as an orange ribbon. The cartoon below the structural model represents the relative orientation of the two RAD51 protomers in the active filament, which obscures the binding site for BRC4. (**B**) Two protomers from our comparative model for the inactive RAD51 filament are displayed with BRC4 using the same colour code as in the previous panel. Rotation of the protomers by ∼15° from their counterparts in the active filament exposes the BRC4 binding site (cartoon). This new conformation is free of apparent atomic clashes, speaking to its plausibility.

However, in our model for the active presynaptic filament of RAD51 bound to the non-hydrolyzable ATP analogue AMP-PNP, the binding site for the Phe-X-X-Ala module is buried in the RAD51 protomer–protomer interface, and therefore likely to be inaccessible for BRC repeat binding. This prompted us to speculate whether changes in protomer–protomer interface geometry associated with the transition from the extended helical conformation of active, ATP-bound filaments to the compact conformation of inactive, ADP-bound filaments might expose this binding site. To test this notion, we structurally aligned a monomer from the crystal structure of inactive, ADP-bound RecA (40) (PDB: 1XMS) to a RAD51 monomer from our active presynaptic filament using Chimera (34). The monomeric structures of the proteins superimpose to within 2Å over a 180 residue region comprising RecA residues 37–268 and RAD51 residues 99–334. As expected, residues 1–99 in the N-terminal region of RAD51 do not correspond to RecA, and were omitted from the analysis. We then used this alignment to create a comparative model for an inactive ADP-bound filament of RAD51 using the MODELLER v9.14 software suite (38,39). The best of 5 possible models was chosen from an assessment of the DOPE statistical potential (41) to represent the inactive ADP-bound filament of RAD51. Supplementary Figure S8 shows the superposition of the inactive RAD51 filament model (6 monomers in red, marked I_A_-I_F_) over our presynaptic filament structure (monomers in green, marked A_A_-A_F_). Interestingly, the inactive filament model predicts that successive RAD51 monomers are rotated by ∼15°, displacing the protomer-protomer interface (Figure 8, compare panel A with B). Whilst the BRC4 peptide from our RAD51-BRC4 monomeric structure (PDB: 1N0W) clashes with the RAD51 protomer-protomer interface in the active RAD51 filament, the relative rotation of RAD51 protomers in the inactive RAD51 filament model exposes the binding site for the Phe-X-X-Ala module from BRC4, enabling free access (Figure 8, compare panel A with B). These observations suggest a hypothetical mechanism wherein changes in RAD51 protomer–protomer interface geometry that occur during the transition from extended, active ATP-bound RAD51 filaments to compact, inactive filaments following ATP hydrolysis may uncover a filament-inhibitory binding site for the Phe-X-X-Ala module from BRC repeats to promote RAD51 filament disassembly.

In addition to effects on RAD51 disassembly, experimental data suggest that the BRC repeats when present in sub-stoichiometric concentrations can *promote* presynaptic RAD51 filament formation (2,5) by mechanisms whose structural basis remains unclear (56). Indeed, we observe that a second module in BRC4 binds to a distinct site on the core catalytic domain of the cyan RAD51 protomer, distant from the protomer–protomer interface in our filament model (Figure 7A and C). The module Leu-Phe-Asp-Glu in BRC4 which engages this second binding site is conserved in other BRC repeats (11,55), and short peptides containing this module alone are capable of binding to RAD51 *in vitro* (55). These considerations raise the possibility that BRC repeat binding to promote assembly of the presynaptic RAD51 filament may occur exclusively via this second binding site, which is compatible with filament formation (Figure 7C). Interestingly, in our filament model the binding of BRC4 to this second binding site is predicted to displace the N-terminal region of the corresponding RAD51 protomer (cyan), suggesting a feature of BRC repeat-bound RAD51 filaments (57).

Collectively, these considerations suggest a hypothetical model for the structural mechanism by which two modules in the BRC repeats of the BRCA2 tumour suppressor engage distinct binding sites on RAD51 filaments to control filament formation and disassembly during different steps in HR. We speculate that the BRC repeats may promote filament formation via interactions involving one conserved module, Leu-Phe-Asp-Glu, whose binding is not expected to perturb the inter-protomer interface (Figure 7C). By contrast, the binding site for a second conserved module, Phe-X-X-Ala, in BRC repeats is positioned at the protomer–protomer interface and possesses filament-inhibitory potential (Figure 7B). This filament-inhibitory binding site is obscured in our structure for the active RAD51 presynaptic filament with 103Å pitch (Figure 7A), but structural modelling suggests that it is uncovered by inter-protomer rotation in more compact, inactive filaments (Figure 8). By analogy, we speculate that the filament-inhibitory binding site may also be accessible in RAD51-dsDNA filaments, which may assume a conformation with decreased pitch (19,58). Thus, changes in inter-protomer geometry induced by decreased filament pitch may potentiate the disassembly of inactive filaments, or selectively inhibit RAD51-dsDNA filament assembly, via the Phe-X-X-Ala module in BRC repeats.

**Figure 9.**
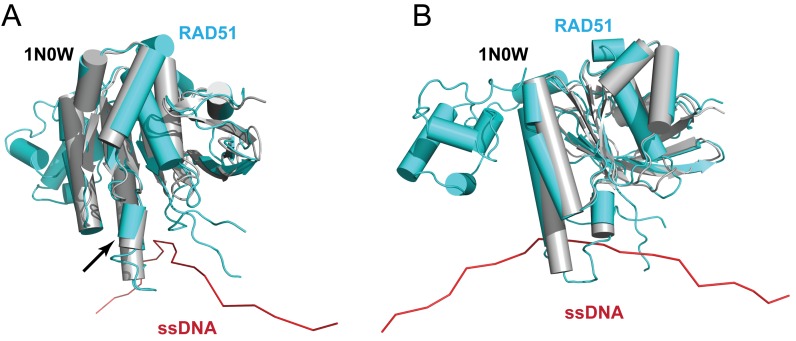
BRC4-bound and ssDNA-bound RAD51. (**A**) The front view of a superposition of a RAD51 monomer from our presynaptic filament model (cyan) on BRC4-bound, N-terminally truncated RAD51 derived from 1N0W (grey). The black arrow highlights the movement of a short α-helix between the two structures. (**B**) The side view of (A).

Superposition (Figure 9A and B) of the core catalytic domain of BRC4-bound monomeric RAD51 (grey) onto a single RAD51 protomer (cyan) from the presynaptic RAD51 filament model on ssDNA (red) reveals that the short α-helix spanning residues Ser223 to Arg229 moves outward away from ssDNA in the filament (arrow in Figure 9A). The helical region is distant from the sites for BRC repeat binding, but lies adjacent to the L2 loop involved in DNA binding, suggesting that its outward displacement may help to accommodate ssDNA in the presynaptic filament.

## SUMMARY

In summary, the findings we report here represent a high-resolution, near-atomic model for a helical nucleoprotein filament of a eukaryotic RAD51 protein bound to DNA resolved by electron cryo-microscopy. Our work enhances structural insights from previously reported structures for bacterial RecA bound to DNA (12), and for different forms of RAD51 without DNA (16), to reveal several novel structural features of potential biological importance. In particular, as discussed in the foregoing, our results suggest a mechanism whereby the RAD51–DNA interaction modulates the helical twist and rise of RAD51 protomers assembled in a filament, help to explain how disease-associated RAD51 mutations affect filament assembly, and rationalize potential structural differences between inhibitory or stabilizing interactions of the BRC repeats of BRCA2 with RAD51 filaments. Moreover, our work exemplifies the power of electron cryo-microscopy in resolving the structure of ordered but anisotropic assemblies of relatively small proteins like RAD51, which are often refractory to crystallographic characterization unless artificially tethered into isomorphic assemblies by the creation of extended fusion proteins (12).

## Supplementary Material

SUPPLEMENTARY DATA

## ACCESSION NUMBERS

Data reported herein have been deposited in the EMDataBank and wwPDB databases with accession codes EMD-8183 (EM map) and 5JZC (PDB coordinates).
