# Supplementary material for: High-resolution structure of the presynaptic RAD51 filament on single-stranded DNA by electron cryo-microscopy
Source: Nucleic Acids Res. 2016 Sep 5;44(19):9017–30. doi: 10.1093/nar/gkw783 (PMC5100573; doi:10.1093/nar/gkw783)
Supplement: SUPPLEMENTARY DATA [file supp_44_19_9017__index.html]

High-resolution structure of the presynaptic RAD51 filament on single-stranded DNA by electron cryo-microscopy — SUPPLEMENTARY DATA 

# High-resolution structure of the presynaptic RAD51 filament on single-stranded DNA by electron cryo-microscopy

## SUPPLEMENTARY DATA

- SUPPLEMENTARY DATA
