# Supplementary material for: High-resolution structure of the presynaptic RAD51 filament on single-stranded DNA by electron cryo-microscopy
Source: Nucleic Acids Res. 2016 Sep 5;44(19):9017–30. doi: 10.1093/nar/gkw783 (PMC5100573; doi:10.1093/nar/gkw783)
Supplement: SUPPLEMENTARY DATA [file supp_gkw783_nar-01990-h-2016-File011.pdf]

High-resolution structure of the presynaptic RAD51 filament on single-stranded DNA by electron cryo-microscopy.

Judith M. Short[1*], Yang Liu[1*], Shaoxia Chen[2], Neelesh Soni[3], M.S. Madhusudhan[3,4], Mahmud K. K. Shivji[1], and Ashok R. Venkitaraman[1]

SUPPLEMENTARY FIGURES AND FIGURE LEGENDS

Supplementary Figure S1. SDS gel and EM micrograph. (A) Coomassie-blue-stained SDS gel of RAD51. (B) A typical micrograph showing filaments of RAD51-bound ssDNA. A short stretch of DNA (72-mer) was selected to reduce bending and prevent duplexing. Frozen hydrated specimens were imaged by a FEI Titan-Krios electron cryo-microscope at 300 keV and recorded on a Falcon detector with the magnification of 104,478x giving the final sampling rate of 1.34Å/pixel.

Supplementary Figure S2. Image processing. The flowchart summarises the image processing of filaments extracted from EM micrograph images. A starting reference model was calculated by a modified version of IHRSR (1), which also determined the helical parameters. RELION (2) was used in conjunction with the IHRSR helical programs hsearch_lorentz and himpose which were run at the end of each 3D classification, 3D refinement and post-processing steps. Motion correction Motioncorr (3) and local CTF correction Gctf (4) software was also used during processing along with particle polishing in RELION. Several iterations of 3D classification and refinement were carried out during the processing.

Supplementary Figure S3. Ramachandran plots of amino acids in the final protein model. Ramachandran plot of amino acids in the final model, which contains three RAD51

and one (18 nt) ssDNA molecule. The red, yellow, and grey regions respectively represent the preferred or allowed regions and outliers.

Supplementary Figure S4. Helical pitch. The helical pitch varies between and sometimes within filaments but averages at 103Å. (A) Two filaments appearing by eye to vary in pitch were selected and their Fourier transforms calculated. The gallery shows left to right each filament with its Fourier transform amplitudes. Each transform shows a clear layer line of Bessel order 1 corresponding to the helical pitch; measurements are indicated on the left and right at 93Å and 105Å. (B) 19 smooth helical models with pitch varying from 76Å - 134Å were created from the IHRSR starting model and low-pass filtered to 100Å. The stack of segments was similarly filtered and aligned to the 19 models. The results shown in the histogram show the pitch spread with most filament segments lying between 95Å and 110Å with the average at 103Å, which agrees precisely with the parameter value at convergence by IHRSR. The emergence of a single model after 3D classification in RELION confirmed that the pitch of the segments included in the reconstruction was contained within a narrow band.

Supplementary Figure S5. Orientation of ssDNA in the density map. PDB coordinates of RecA-bound ssDNA (5) were converted into EM density using SPIDER (6), then low-pass filtered to 5Å. The inner region of our RAD51_ssDNA map was masked with a cosine-edged cylinder of radius corresponding to the average diameter of ssDNA (7Å) and compared to the converted RecA_ssDNA density in Chimera (7). The fit between the RecA-bound ssDNA density and the cylindrically masked area of our HsRAD51 map as estimated by Chimera returned a correlation coefficient of 0.95, allowing us to overlay the pdb coordinates of the DNA from the RecA structure directly into our map. The transparent rendering of the protein backbone (grey) reveals ssDNA (blue) threaded along the filament axis.

Supplementary Figure S6. ssDNA-protein contacts.

A) Coordinates from a single RecA monomer with its bound ssDNA were extracted from the deposited PDB file (5). B) PDB coordinates derived from our presynaptic filament model (Figure 3) representing ssDNA with a single RAD51 monomer were aligned to the RecA coordinates for comparison. The contact regions are disordered in both the RecA and RAD51 filament structures, so it is not possible to precisely visualise details. However, the 3 black arrows indicate the regions where ssDNA-protein contacts most likely occur in the RAD51 structure. The two loop regions L1 and L2 are indicated by blue arrows.

Supplementary Figure S7. The protomer-protomer interface.

A) To investigate the possibility of an alternating conformation at the interface between protomers, we used our refined map as a reference model for 3D classification of EM images in RELION without imposing helical symmetry, and iterated 110 times. The most populated (>90%) model (red) was visualised in Chimera, before a second copy of this model (blue) was fitted to the first model then axially translated and rotated by one protomer before overlaying just as before. No movement is evident between the two large α helical regions (arrowed) in the overlaid human RAD51 filament models. B) Dimer symmetry was imposed on the refined map before 3D classification and refinement and again following both classification and refinement. No widening of the two α–helical regions was observed here either, suggesting that the protomer-protomer interface exists in only a single, invariant geometry.

Supplementary Figure S8. Re-orientation of RAD51 subunits during transition to an inactive filament may expose a BRC repeat binding site.

Comparative modelling of the active presynaptic RAD51 filament using inactive ADP-bound RecA as a template was carried out as described in Methods to create a hypothetical model for an inactive RAD51 filament. The figure shows the superposition of the inactive RAD51 filament model (6 monomers in red, marked I_A-I_F) over our presynaptic filament structure (monomers in green, marked A_A-A_F). The main chains of the D monomeric units of both filaments are represented in ribbons while the other monomers are shown in surface representation.

REFERENCES

1. Egelman, E.H. (2000) A robust algorithm for the reconstruction of helical filaments using single-particle methods. Ultramicroscopy, 85, 225-234.
2. Scheres, S.H. (2012) RELION: implementation of a Bayesian approach to cryo-EM structure determination. J Struct Biol, 180, 519-530.
3. Li, X., Mooney, P., Zheng, S., Booth, C.R., Braunfeld, M.B., Gubbens, S., Agard, D.A. and Cheng, Y. (2013) Electron counting and beam-induced motion correction enable near-atomic-resolution single-particle cryo-EM. Nat Methods, 10, 584-590.
4. Zhang, K. (2016) Gctf: Real-time CTF determination and correction. J Struct Biol, 193, 1-12.
5. Chen, Z., Yang, H. and Pavletich, N.P. (2008) Mechanism of homologous recombination from the RecA-ssDNA/dsDNA structures. Nature, 453, 489-484.
6. Frank, J., Radermacher, M., Penczek, P., Zhu, J., Li, Y., Ladjadj, M. and Leith, A. (1996) SPIDER and WEB: processing and visualization of images in 3D electron microscopy and related fields. J Struct Biol, 116, 190-199.
7. Pettersen, E.F., Goddard, T.D., Huang, C.C., Couch, G.S., Greenblatt, D.M., Meng, E.C. and Ferrin, T.E. (2004) UCSF Chimera--a visualization system for exploratory research and analysis. Journal of computational chemistry, 25, 1605-1612.

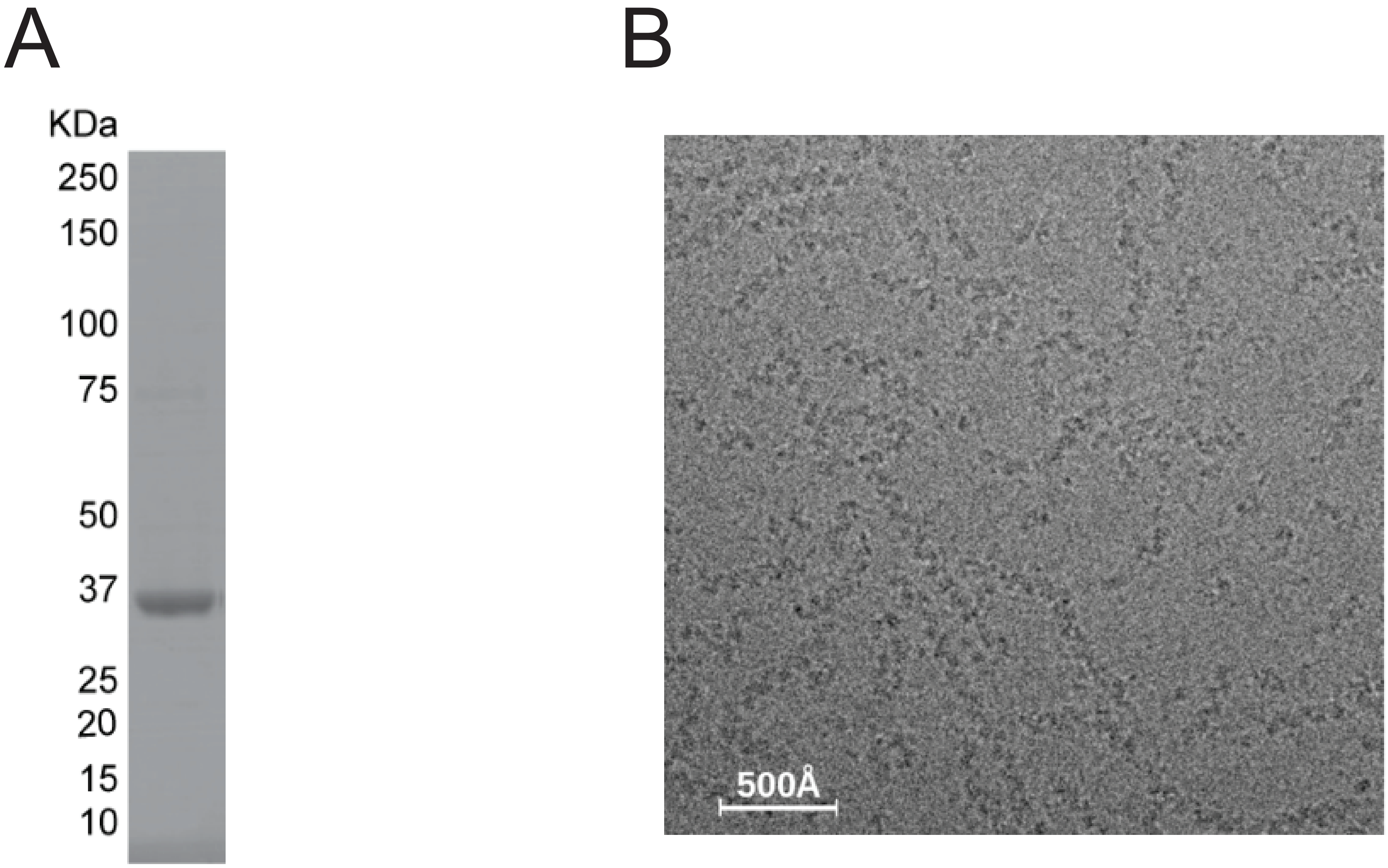

Supplementary Figure S1

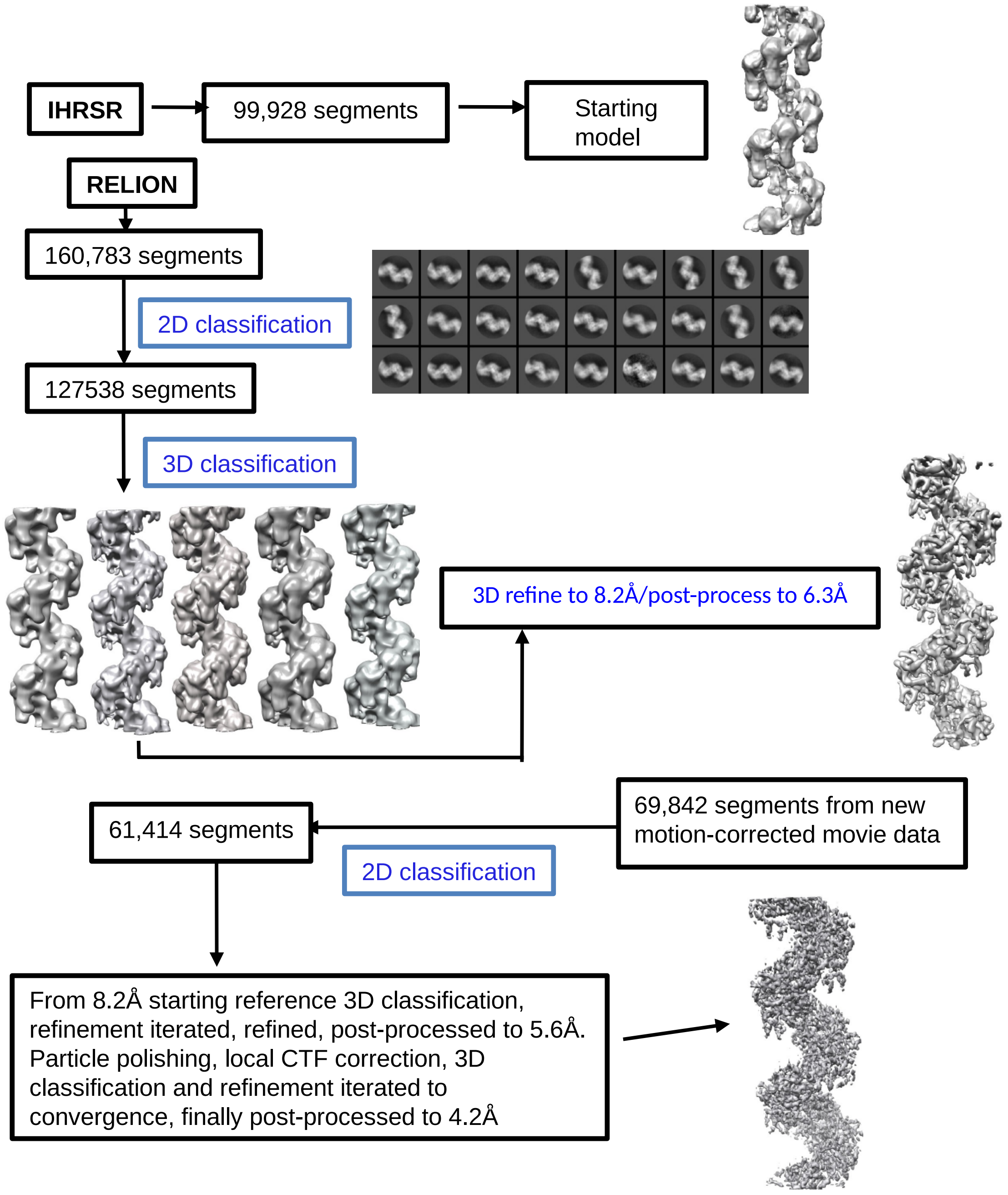

Supplementary Figure S2

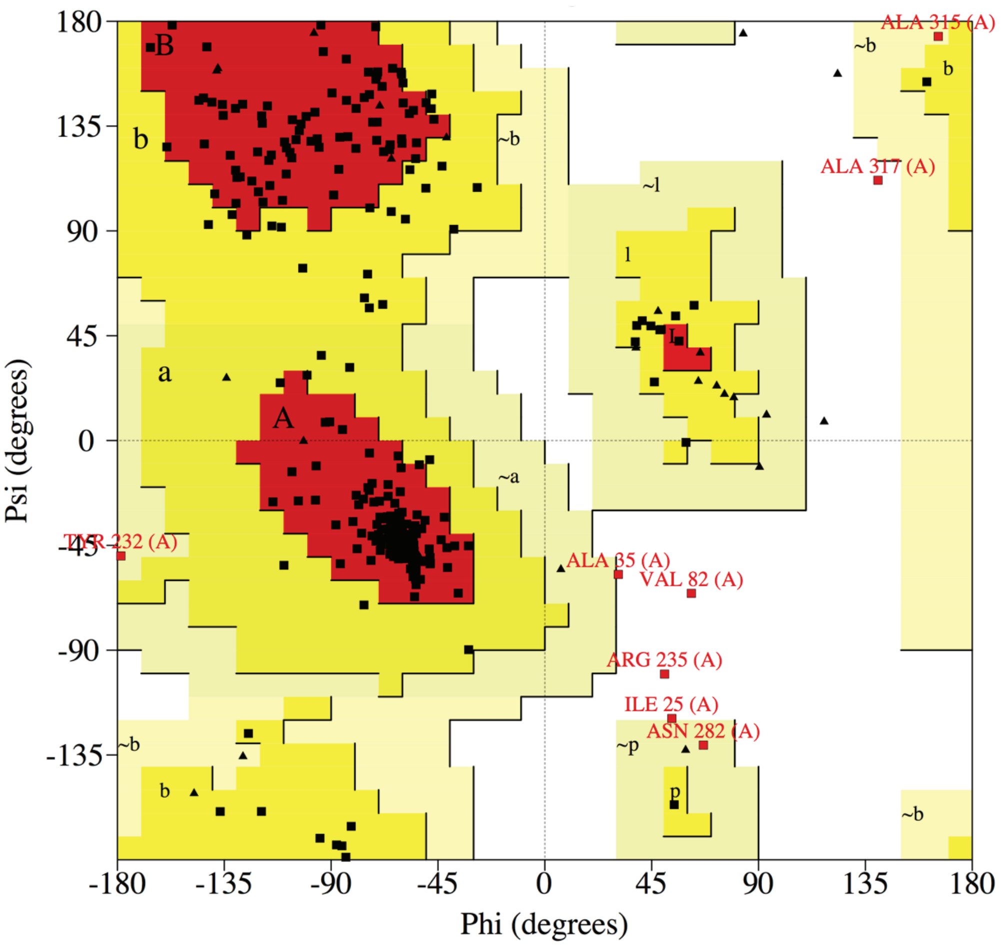

Plot statistics

Residues in most favoured regions [A,B,L]	218	79.9%
Residues in additional allowed regions [a,b,l,p]	47	17.2%
Residues in generously allowed regions [~a,~b,~l,~p]	3	1.1%
Residues in disallowed regions	5	1.8%
	----	------
Number of non-glycine and non-proline residues	273	100.0%
Number of end-residues (excl. Gly and Pro)	3	
Number of glycine residues (shown as triangles)	26	
Number of proline residues	10	

Total number of residues	312	

Supplementary Figure S3

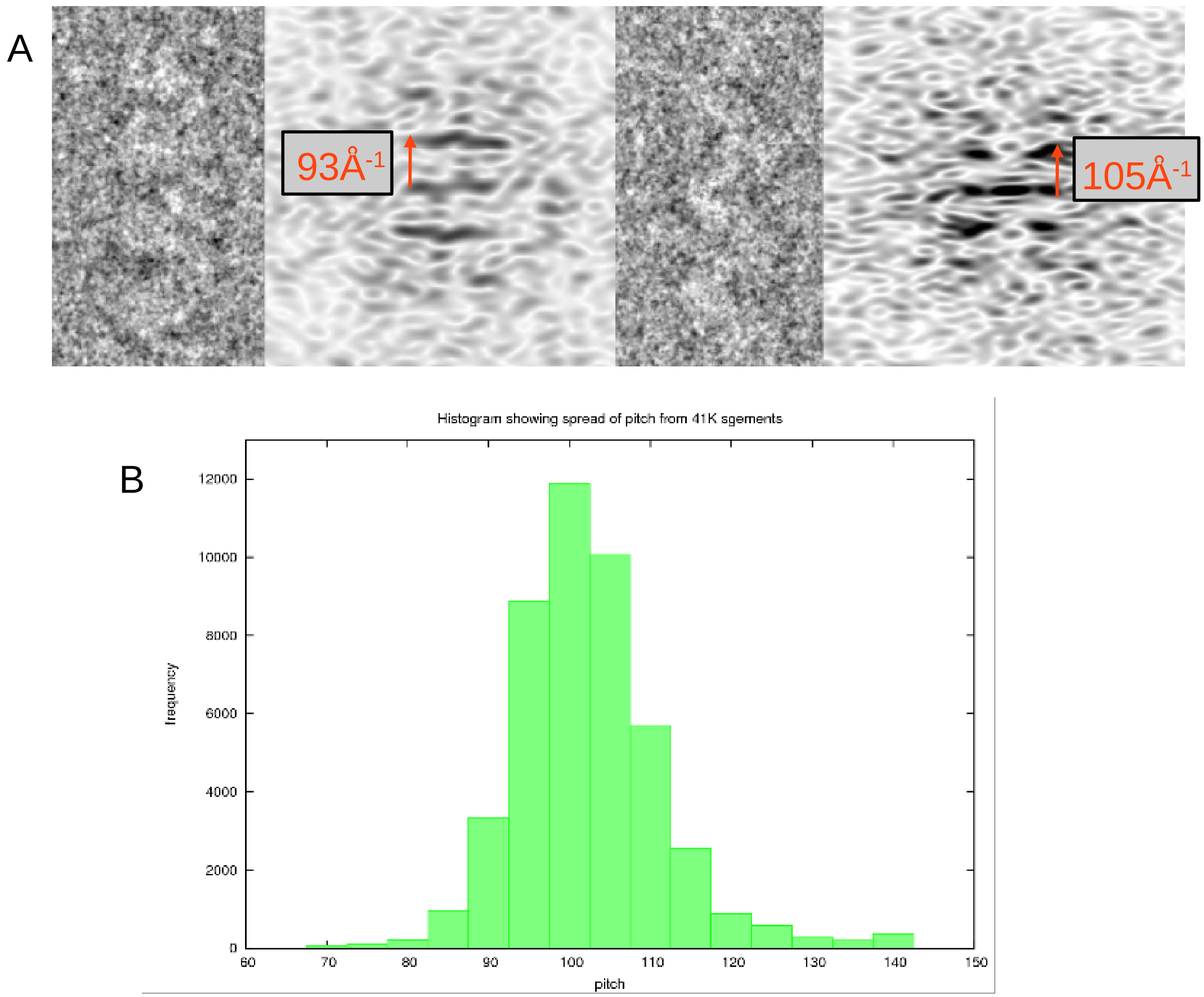

Supplementary Figure S4

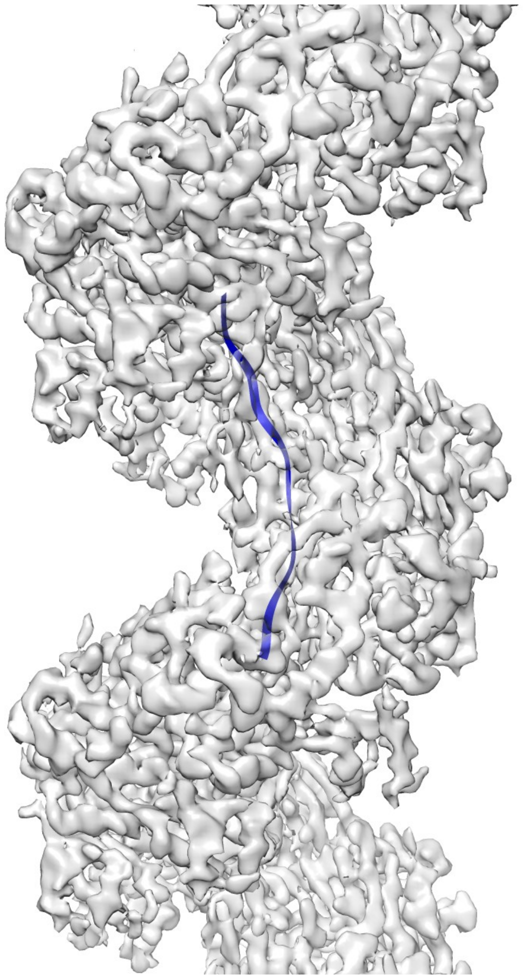

Supplementary Figure S5

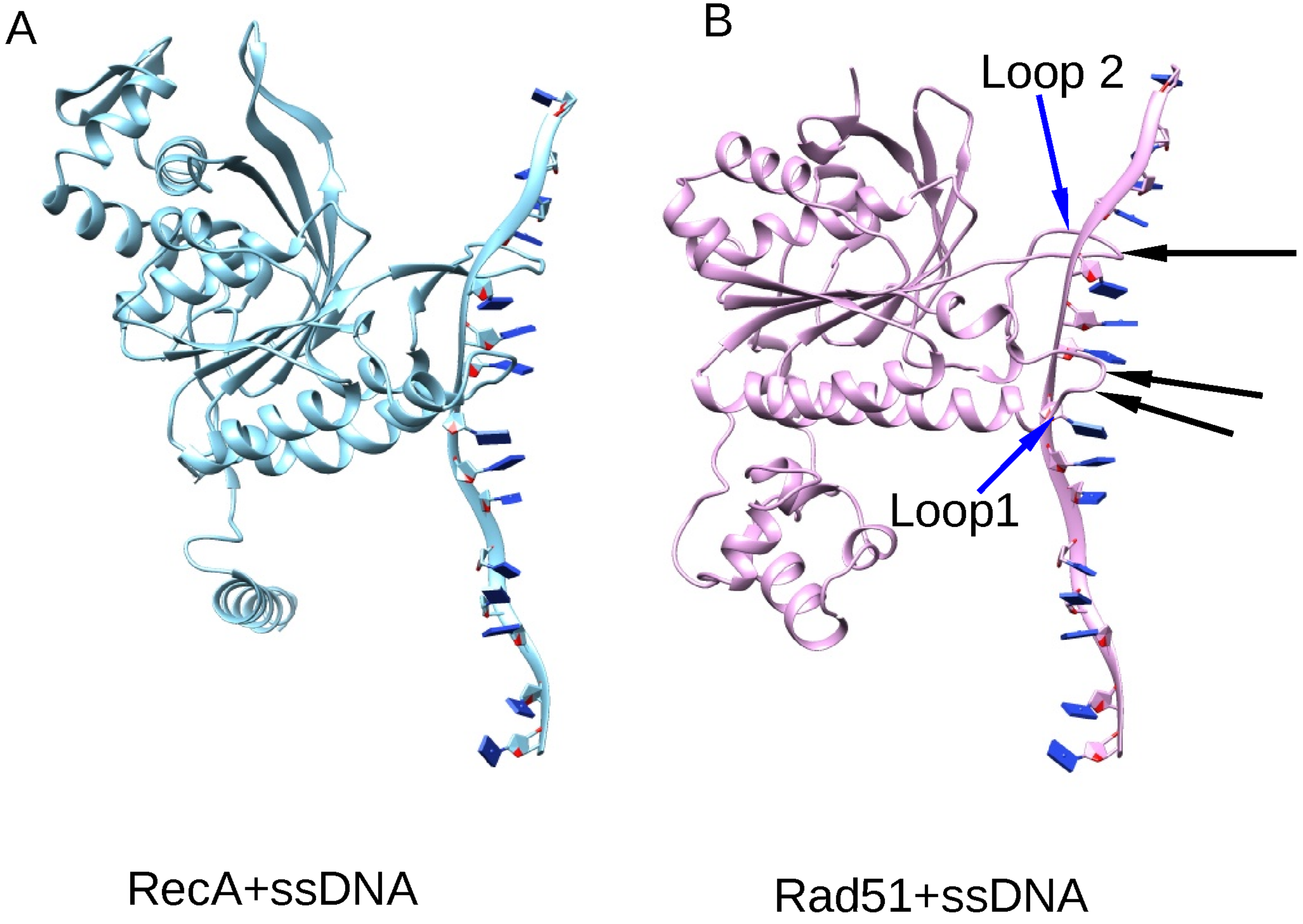

Supplementary Figure S6

A

B

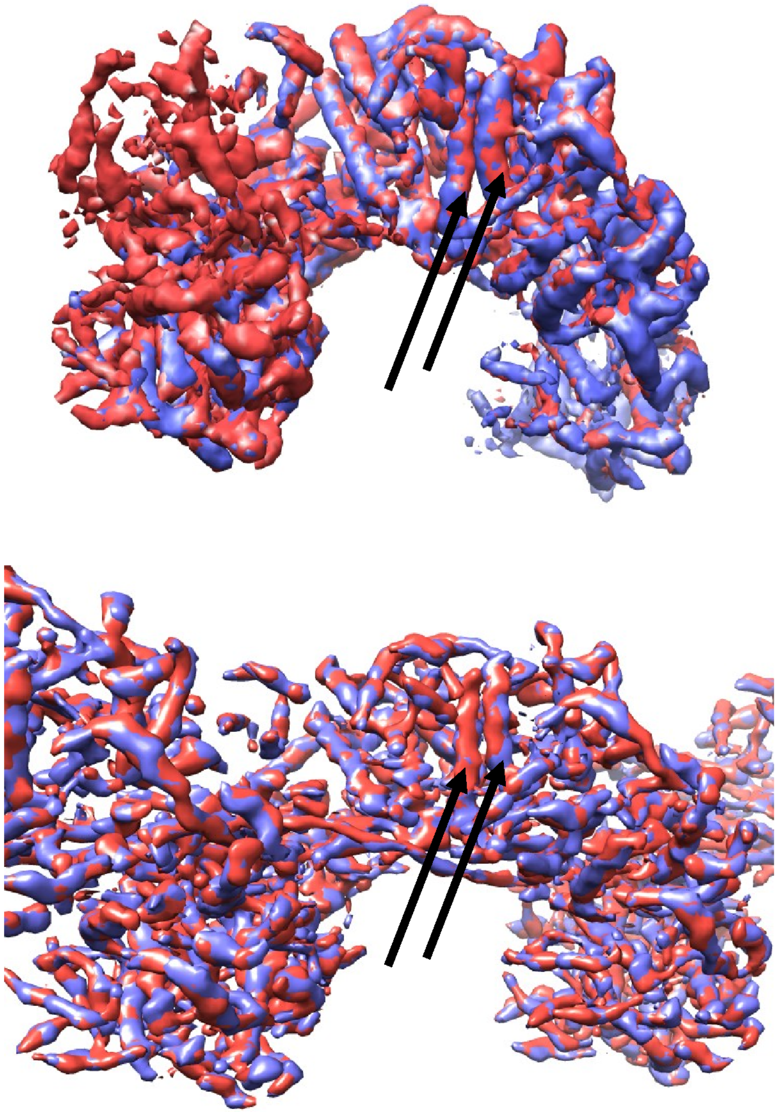

Supplementary Figure S7

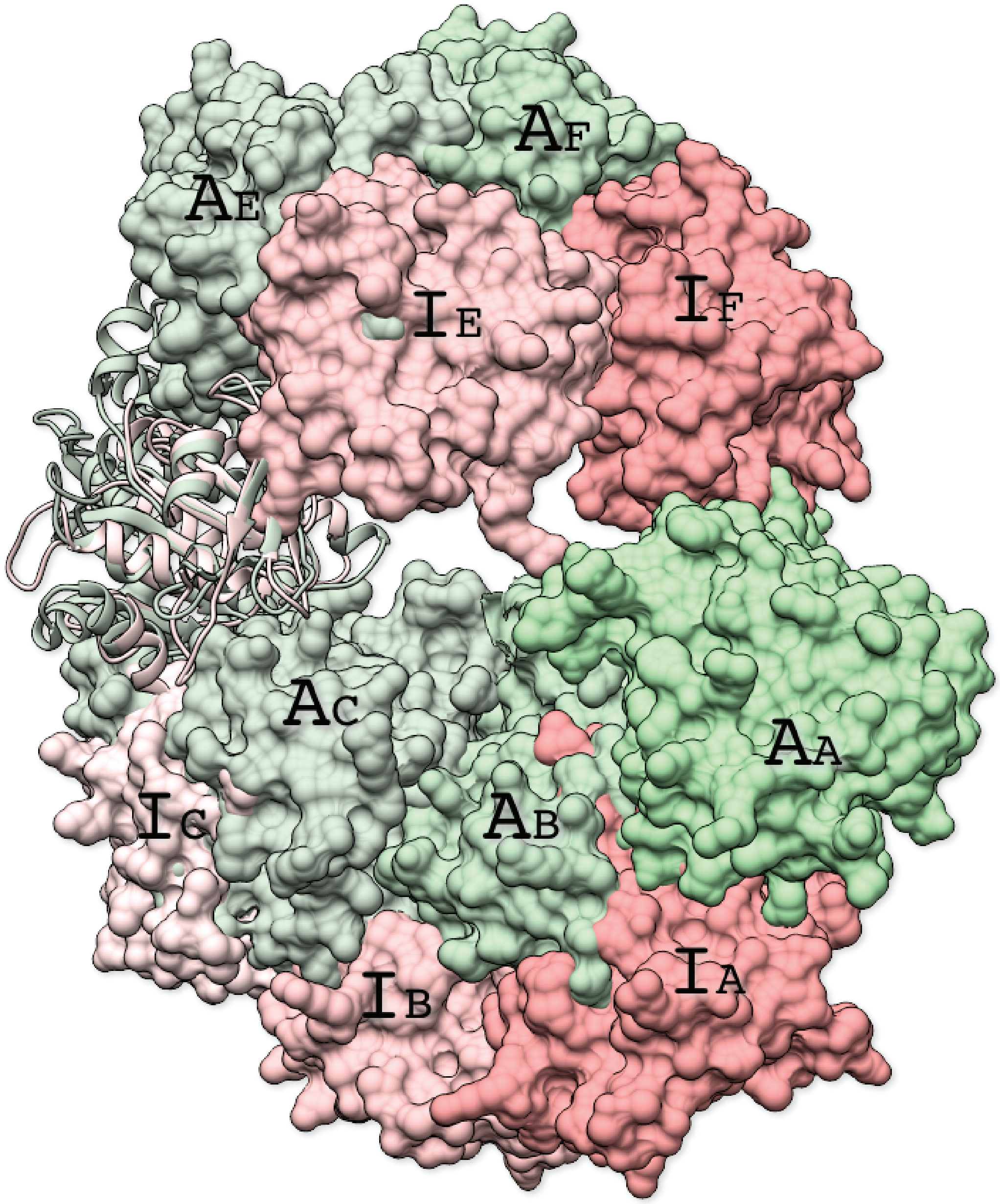

Supplementary Figure S8
